# Numerical investigation of residual stresses in thin-walled additively manufactured structures from selective laser melting

**DOI:** 10.1016/j.heliyon.2023.e19385

**Published:** 2023-08-24

**Authors:** Nissar Ahmed, Imad Barsoum, Rashid K. Abu Al-Rub

**Affiliations:** a Advanced Digital & Additive Manufacturing Center, Khalifa University of Science and Technology, 2533 Abu Dhabi, United Arab Emirates; b Department of Mechanical Engineering, Khalifa University of Science and Technology, 2533 Abu Dhabi, United Arab Emirates

**Keywords:** Thin-walled structures, Residual stresses, Additive manufacturing, Selective laser melting, Finite element modeling

## Abstract

Selective laser melting (SLM), a metal laser powder bed fusion additive manufacturing method, involves several cycles of very high temperature gradient heating and cooling during the solidification of each layer, which can cause the accumulation of detrimental residual stresses in the 3D printed structure. This work uses a thermo-mechanical computational modeling approach to investigate the formation of residual stresses in thin-walled structures and also investigates the effects of varying taper on the evolution of residual stress profiles of the build. Three material grades; namely, Titanium alloy (Ti64), Stainless steel (SS316L) and Inconel (IN718) have been used for this study. The results show that varying taper thickness up to a certain value has a considerable effect on residual stress evolutions in thin-wall structures, however, beyond a certain value of the taper level, the residual stresses are observed to converge. Also, it is observed that the tensile stresses at the edges of the wall are almost equal or exceed the yield stress of the materials. Among the three material grades considered, the magnitude of residual stress was higher in Ti64 and the stresses are dominant in the build direction. The simulation framework is also applied to analyze the effect of residual stresses on the mechanical properties of complex thin-wall structures such as TPMS (Triply Periodic Minimum Surfaces) lattice structure, using Schwarz Primitive (SP) as a case study. It is observed that the residual stresses lower the effective elastic properties of the lattice structures by 6% ∼ 10% for the three material grades but has no effect on the effective plastic behavior of the material.

## 1 Introduction

For past couple of decades, the metal laser powder bed fusion selective laser melting (SLM) process has been a widely used additive manufacturing (AM) technique for manufacturing functional parts in industries such as aerospace, automotive and biomedical. Fabrication of complex geometries is now being enabled using the SLM process, which are not possible with conventional manufacturing methods,eg. precision thin-walled metal structures below 0.5 mm thickness are difficult to fabricate with traditional machining methods due to the low rigidity and the cutting force [1]. However, despite the freedom of design for AM process, there are still obstacles in its wide-scale adoption due to numerous AM parameters involved that must be understood properly in order to manufacture high quality parts [2,3]. The layer-by-layer nature of SLM process sets up extremely large thermal gradients in the build parts that result in the formation of residual stresses and may lead to dimensional inaccuracies, part distortions and crack formation [[4], [5], [6]]. High-performance thin-wall structures made of superalloys are often needed for aerospace structures and medical implants Thin-wall sections are also used in heat exchangers to help heat flow. However, the criticalities involved in SLM process, especially the challenges associated with controlling distortion due to the thermal stress during the fabrication needs to be addressed [7].

The investigation of residual stresses has been a key area of research interest within the AM community aimed mainly at understanding its profile evolution mechanisms and its mitigation in built parts [[8], [9], [10], [11]] using experimental and numerical approaches. Maly et al. [12] studied the effect of process parameters such as laser power, scanning speed and preheating powder bed on residual stresses of Ti6Al4V samples produced by SLM, and found that the preheating powder bed to high temperature (∼550 °C) was the most effective parameter in reducing residual stress. Mugwagwa et al. [13] in their study found that the influence of various process parameter has to be simultaneously studied to mitigate residual stress and distortions in parts, in which they investigated the combination of laser power, scan speed and layer thickness. Others [[14], [15], [16]] have studied the influence of various scan strategies on residual stress evolution in SLM process and found that adopting effective scanning paths, which results an even distribution of heat in the layer, leads to a considerable reduction in the magnitude of residual stress.

Several studies have adopted fairly complex computational methods to evaluate the residual stress formation in SLM thin-walled metal parts. Chen et al. [17] used a three-dimensional (3D) indirect sequentially coupled thermo-mechanical finite element model (FEM) to predict residual stress distribution in thin-walled structures, and found that residual stresses increase with increasing laser power and decrease with increasing printing scan speed. Mukerjee et al. [18] incorporated fluid flow in their FEM models by considering the convective heat transfer in the molten pool and thus accurately predicting the residual stress formation and distortions. Their results suggest reducing the layer thickness to mitigate residual stress formations in Ti64 and IN718 thin-walled specimens. Waqar et al. [19] used a thermo-mechanical approach and found that preheating the baseplate and powder bed remarkably reduced the residual stress formations in SS316L specimens. Li et al. [20] investigated numerically the effect of scan length on the shrinkage of the thin-walled parts, and found that there is an increase in deviation due to increasing scan lengths and suggested a suitable range of scan lengths for minimum deviation of the part. The thermo-mechanical modeling approach has also been used to model other AM processes such as electron beam melting (EBM) [21,22], for example Abdullah et al. [23] used FEM to predict the temperature distribution, distortion, and residual stresses as well studied the influence of process parameters in fabricating thin-walled Ti64 parts, where the simulated pattern of the distortion and temperature profiles were found to be in close agreement with the experimental data.

As it is computationally expensive to simulate the thousands of actual layers of powder deposition occurring in SLM, a numerical efficient lumped model approach in which a single layer is representative of several actual layers of deposition may be used. This modeling method has been commonly used in simulation of the welding process [24], and has been adopted by researchers to investigate part distortions and residual stress evolutions in SLM parts as well. Lu et al. [25] and Chen et al. [26] used a lumped layer approach in their FEM model to study thin-walled structures of various configurations and to minimize distortions. Similarly, lumped layer-wise activation method was used by An et al. [27] in their FEM simulations to accurately predict the residual stress patterns in curved thin-walls, and found residual stresses to be tensile near the free edges and compressive in the middle region of the walls. Hence, lumped modeling approach can be adopted to accelerate the simulation time as well as accurately predict the residual stresses and distortions in parts.

Tapered (i.e. variable wall thickness) thin-walled structures are versatile design elements that offer several benefits in various industries including weight reduction, high energy absorption, efficient heat transfer and design flexibility [[28], [29], [30]]. A simulation framework which can be used for investigating the residual stress formation in tapered thin walls fabricated using the AM process is rarely investigated. In the current study, a thermo-mechanical based FEM model that can provide insight into residual stress states in thin-walls as well as the effect of taper dimensions on stress evolutions is investigated. The taper effect is studied by varying the bottom thickness of a thin wall, i.e. the region attached to the baseplate. The numerical study is conducted using three different material grades; Ti64, SS316L and IN718, which are commonly used metal alloys in AM. The findings of this study may be useful to further carry out parametric studies to reduce residual stresses saving both time and costly experiments.

Moreover, TPMS (Triply Periodic Minimum Surface) lattices [31] have recently been receiving increasing interest in investigating their mechanical properties, however without considering effects of residual stresses generated during the metal laser powder-bed fusion (LPBF) additive manufacturing process when various types of metal powder are used. Thus, as an application case study of the current simulation framework, the Schwarz Primitive (SP) thin-walled TPMS lattice structure made of various types of metals has been used to study the effect of residual stresses on its overall effective mechanical properties.

## 2 Computational modeling framework

### 2.1 Constitutive equations

**Fig. 1:**
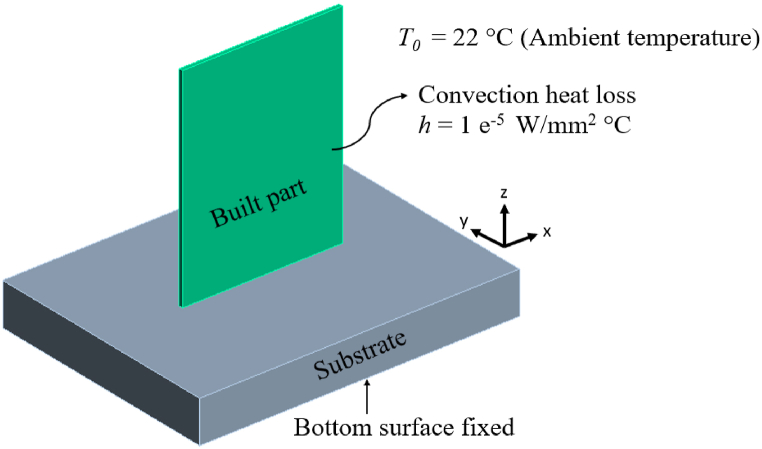
Initial and boundary conditions of the simulation model.

For the AM process simulation, the material is assumed to be homogenous with isotropic thermo-mechanical properties. Thus, the transient temperature distribution *T* (*x, y, z, t*) throughout the build during the SLM process is governed by the following heat equation (1):

(1) $$k\;\nabla^{2}T+Q=\rho \;C_{p}\frac{\partial T}{\partial t}$$

where *C*_*p*_ is the temperature dependent specific heat capacity, *ρ* is the density, *T* is the temperature, *t* is the time, *Q* is the internal heat generation rate and *k* is the temperature dependent thermal conductivity of the base material. The initial and final (e.g. cooling stage) thermal conditions for temperature distribution throughout the powder bed at the start of the process (*t* = 0) is 22 °C and the end (*t* = ∞), i.e. cooling stage is at 22 °C, which is the reference/ambient temperature *T*_*0.*_ During the AM build formation, base of the substrate is preheated to a temperature for e.g.: 100 °C i.e. for the time interval 0 < *t* < ∞ and brought back to room temperature during the build cooling phase (*t* = ∞). All the other surfaces are assumed to be under heat loss to the surrounding gas by free air convection with a heat transfer coefficient *h* = 10^−5^ W/mm^2^ °C [32] used in the model. The simulation model with associated initial and boundary condition of the model is illustrated in Fig. 1.

The large thermal gradients in SLM, due to layer-by-layer laser melting process, result in the accumulation of residual stresses and strains during the solidification process. In the mechanical finite element analysis, the temperature results from the transient thermal analysis step are used as inputs. Assuming infinitesimal strains, the stress-strain relationship in the build part is defined in equation (2):

(2) $$\left\{\sigma \right\}=\left[D\right]\left\{\varepsilon^{e}\right\}\text{,}$$

where {*σ*} is the stress vector, [*D*] is the elasticity stiffness matrix, and {ε ^e^} is the elastic strain vector. The solid-state phase change in the material is not considered, using a simplified elastic-plastic hardening model which is often used in thermo-mechanical models [ [33,34]], {*ε* ^*e*^} can be expressed as following in equation (3):

(3) $$\left\{\varepsilon^{e}\right\}=\left\{\varepsilon \right\}-\left\{\varepsilon^{p}\right\}-\left\{\varepsilon^{t}\right\}$$

where, {*ε*}, {ε ^p^}, and {ε ^t^} are the total strain, plastic strain, and thermal strain vectors, respectively. Assuming an isotropic material, stress–strain relationship from equation (3) can be written in a component form using Cartesian *x*, *y*, and *z* coordinate system as follows in equations (4), (5), (6):

(4) $$\varepsilon_{x}=\frac{1}{E}\;\left[\sigma_{x}-\nu \left(\sigma_{y}+\sigma_{z}\right)\right]+\varepsilon_{x}^{p}+\varepsilon^{t}$$

(5) $$\varepsilon_{y}=\frac{1}{E}\;\left[\sigma_{y}-\nu \left(\sigma_{x}+\sigma_{z}\right)\right]+\varepsilon_{y}^{p}+\varepsilon^{t}$$

(6) $$\varepsilon_{z}=\frac{1}{E}\;\left[\sigma_{z}-\nu \left(\sigma_{x}+\sigma_{y}\right)\right]+\varepsilon_{z}^{p}+\varepsilon^{t}$$

(7) $$\gamma_{xy}=\frac{\tau_{xy}}{G}+\gamma_{xy}^{p},\gamma_{xz}=\frac{\tau_{xz}}{G}+\gamma_{xz}^{p},\gamma_{yz}=\frac{\tau_{yz}}{G}+\gamma_{yz}^{p}$$

where *E* is the young's modulus, *G* is shear modulus, ν is Poisson's ratio, and ε is the normal strain. Equation (7) represents the relation between the shearing strain components γ_*xy*_, γ_*yz*_ and γ_*yz*_, and the shear stress components *τ*_*xy*_, *τ*_*yz*_ and *τ*_*zx*_. The thermal strain component arising due to volume change caused by temperature variations can be expressed in following equation (8):

(8) $$\varepsilon^{t}=\alpha_{e}\Delta T=\alpha_{e}\left(T-T_{0}\right)$$

where *α*_*e*_ is the coefficient of thermal expansion, and *T*_*0*_ is the reference temperature with respect to time at *t* = 0. The deviatoric stresses according to Prandtl-Reuss equation of plasticity can be represented in Equations (9), (10):

(9) $$\frac{d\varepsilon_{x}^{p}}{{\sigma ’}_{x}}=\frac{d\varepsilon_{y}^{p}}{{\sigma ’}_{y}}=\frac{d\varepsilon_{z}^{p}}{{\sigma ’}_{z}}=\frac{d\gamma_{xy}^{p}}{\tau_{xy}}=\frac{d\gamma_{yz}^{p}}{\tau_{yz}}=\frac{d\gamma_{zx}^{p}}{\tau_{zx}}=\frac{d\lambda }{\sigma_{e}}\text{,}$$

(10) $${\sigma ’}_{x}=\sigma_{x}-\sigma_{m},{\sigma ’}_{y}=\sigma_{y}-\sigma_{m},{\sigma ’}_{z}=\sigma_{z}-\sigma_{m}\text{,}$$

where *σ′*_*x,*_ *σ′*_*y*_ and *σ′*_*z*_ are the deviatoric stresses in *x*, *y* and *z* directions, respectively, *dλ* is the instant positive constant of proportionality, which is interpreted as the rate of equivalent plastic strain, and *σ*_*m*_ refers to the mean stress represented in equation (11):

(11) $$\sigma_{m}=\frac{\sigma_{x}+\sigma_{y}+\sigma_{z}}{3}\text{,}$$

Substituting the values of $\varepsilon_{x}^{p}$, $\varepsilon_{y}^{p}$ and $\varepsilon_{z}^{p}$ and $\varepsilon_{t}$ in equations (4), (5), (6), (7), the resultant equations can be stated as follows in equation (12), (13), (14), (15) respectively:

(12) $$\varepsilon_{x}=\frac{1}{E}\left[\sigma_{x}-\nu \left(\sigma_{y}+\sigma_{z}\right)\right]+\int \left(\frac{{\sigma ’}_{x}}{\sigma_{e}}\right)d\lambda +\alpha_{e}\Delta T\text{,}$$

(13) $$\varepsilon_{y}=\frac{1}{E}\left[\sigma_{y}-\nu \left(\sigma_{x}+\sigma_{z}\right)\right]+\int \left(\frac{{\sigma ’}_{y}}{\sigma_{e}}\right)d\lambda +\alpha_{e}\Delta T\text{,}$$

(14) $$\varepsilon_{z}=\frac{1}{E}\left[\sigma_{zy}-\nu \left(\sigma_{x}+\sigma_{y}\right)\right]+\int \left(\frac{{\sigma ’}_{z}}{\sigma_{e}}\right)d\lambda +\alpha_{e}\Delta T\text{,}$$

(15) $$\gamma_{xy}=\frac{\tau_{xy}}{G}+\int \left(\frac{\tau_{xy}}{\sigma_{e}}\right)d\lambda ,\gamma_{xz}=\frac{\tau_{xz}}{G}+\int \left(\frac{\tau_{xz}}{\sigma_{e}}\right)d\lambda ,\gamma_{yz}=\frac{\tau_{yz}}{G}+\int \left(\frac{\tau_{yz}}{\sigma_{e}}\right)d\lambda$$

(16) $$\sigma_{e}=\sqrt{{\frac{1}{2}[\left(\sigma_{x}-\sigma_{y}\right)}^{2}+{\left(\sigma_{y}-\sigma_{z}\right)}^{2}+{\left(\sigma_{z}-\sigma_{x}\right)}^{2}+6\left(\tau_{xy}^{2}+\tau_{yz}^{2}+\tau_{zx}^{2}\right)}$$

Finally, the von-Mises equivalent stress *σ*_*e*_ is expressed in equation (16).

### 2.2 Materials

Temperature-dependent physical and thermal properties of solid base Ti64, SS316L and IN718 including density *ρ*, specific heat *C*_*p*_ and thermal conductivity *k* are used as input properties to perform the transient thermal analysis. For the mechanical analysis, the temperature dependent elastic modulus *E*, yield strength *σ*_*y*_, Poisson's ratio *ν*, plastic tangent modulus *E*_*T*_ and coefficient of thermal expansion *α* are used.

**Fig. 2:**
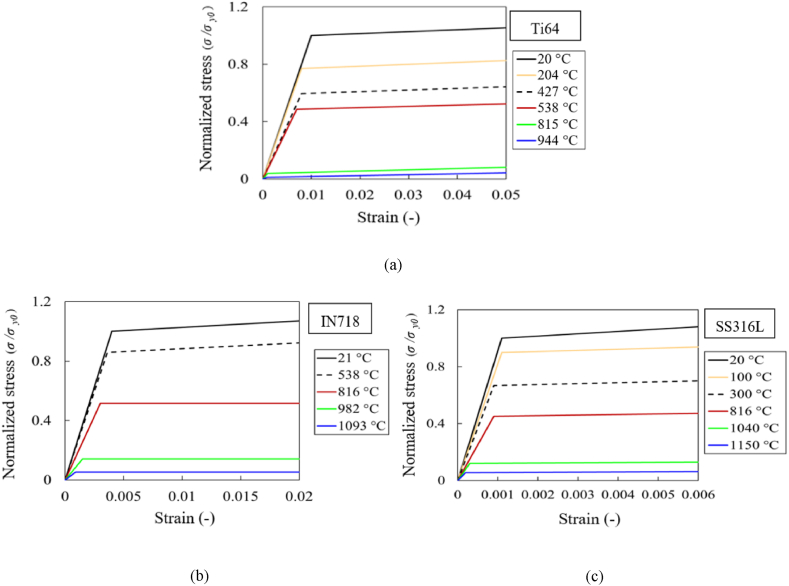
Normalized temperature-dependent stress-strain curves for material grades: (a) Ti64, (b) IN718, and (c) SS316L [32].

**Fig. 3:**
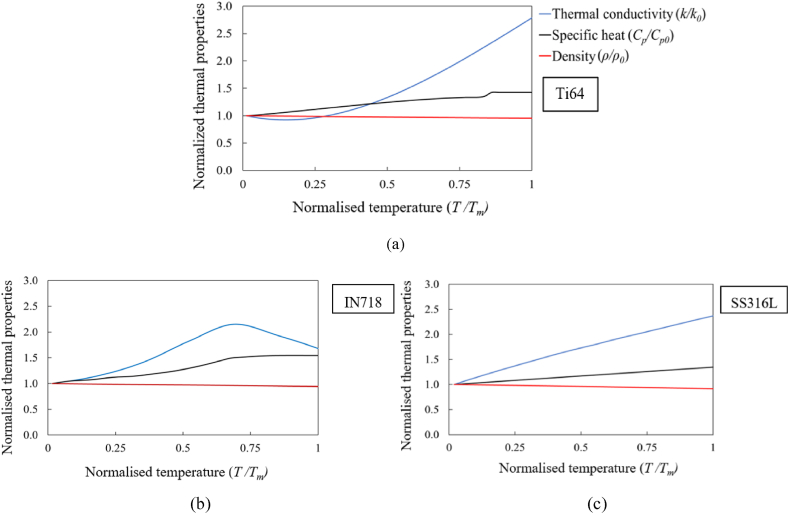
Normalized temperature-dependent thermal and physical properties for material grades: (a) Ti64, (b) IN718, and (c) SS316L [32].

**Fig. 4:**
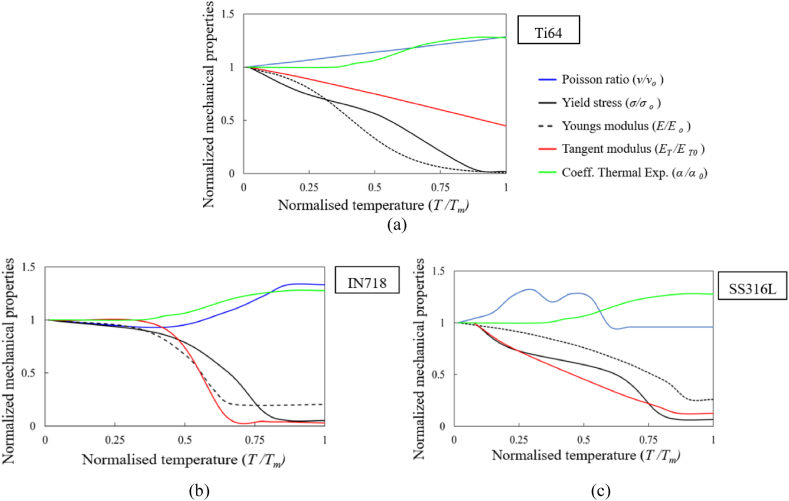
Normalized temperature-dependent mechanical properties for material grades: (a) Ti64, (b) IN718 and (c) SS316L [32].

The stress-strain curves of material grades Ti64, SS316L and IN718 at various temperatures are shown in Fig. 2a–c [32] pertaining to stress normalized with the yield stress at room temperature. The evolution of the temperature-dependent thermal and mechanical properties are shown in Fig. 3a–c [32] and Fig. 4a–c [32] respectively, where the properties are normalized with their corresponding value at room temperature and the temperature is normalized with the melting temperature value of corresponding material grade Ti64 (*T*_*m*_ = 1605 °C), SS316L (*T*_*m*_ = 1370 °C) and IN718 (*T*_*m*_ = 1260 °C). The corresponding properties at room temperature for Ti64, SS316L and IN718 material grades are provided in Table 1.

**Table 1:** Physical, mechanical, and thermal properties of the base material for Ti64, IN718, and SS316L at room temperature (*T*_*0*_) [32].

Property	Ti64	IN718	SS316L
Density *ρ*_*0*_ (kg/m3)	4405	8220	7954
Specific heat *C*_*p0*_ (J/kg °C)	582	421	498
Conductivity *k*_*0*_ (W/m °C)	8.11	11.9	13.44
Initial yield strength σ_*0*_ (MPa)	1098	648	225
Poisson ratio *ν*_*0*_	0.32	0.30	0.25
Thermal expansion *α*_*0*_ (°/C)	8.9 E−06	1.44 E−05	1.47 E−05
Youngs Modulus *E*_*0*_ (MPa)	107	165	195
Tangent Modulus *E*_*T0*_ (GPa)	1332	648	209
Diffusivity *α*_*d0*_ (m^2^/s) × 10^−6^	3.16	3.37	3.39

### 2.3 Finite element model

**Fig. 5:**
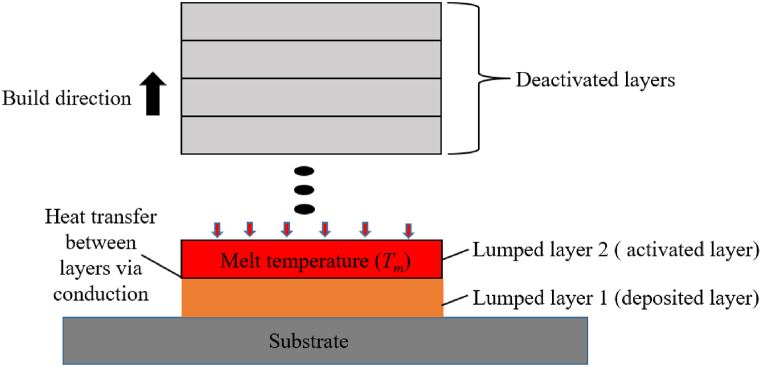
Schematic diagram of the lumped layer approach used in the AM simulation.

**Fig. 6:**
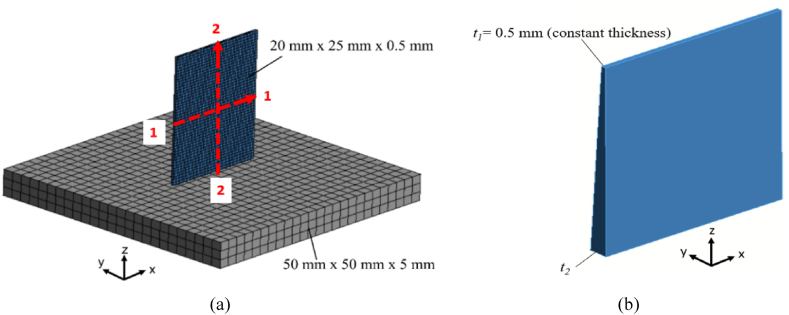
(a) FEM model of a thin-wall on a substrate showing the path of measurement for residual stress components of the plate i.e. along the width (path 1–1) and height (path 2–2) of the wall, (b) wall taper achieved by varying the bottom thickness (*t*_*2*_*)* while top thickness (*t*_*1*_) is kept constant.

To study the residual stresses generated in thin-walled structures the base geometry dimensions considered and the FEM model as shown in Fig. 6a is adopted. The thin plate is of dimensions 20 mm × 25 mm × 0.5 mm, while baseplate of dimensions 50 mm × 50 mm × 10 mm is considered. The paths of residual stress measurements, i.e. path 1–1 across the width and path 2–2 along the height, are indicated by the red dotted lines in Fig. 4a. The effect of taper on residual stresses is investigated by varying the bottom thickness (*t*_*2*_ = 1 mm, 1.5 mm, 2.0 mm and 2.5 mm) while the top thickness (*t*_*1*_ = 0.5 mm) is kept constant as shown in Fig. 6b. To predict the evolution of residual stresses during the SLM process, the indirect sequentially coupled thermal–mechanical analysis method is adopted [35,36]. Ansys-WB is used, where the AM simulation tool is used to perform a thermal analysis followed by a quasi-static elastic-plastic mechanical analysis. The transient thermal analysis first records the temperature histories of elements activated layer-wise using the element birth and death technique, until the build is cooled to room temperature (e.g., 22 °C). The transient mechanical analysis uses the thermal histories as input to evaluate the stresses by activating layers in a similar pattern as in the thermal analysis so that the final residual stress state at the cooling of the build is obtained. As the temperature histories of each adjacent physical layer are similar, the lumped layer approach is used in which the activation of each finite element layer accounts for the deposition of several physical layers of actual metal powder at once, saving computational time and memory resources. The AM simulation employs the implicit solver of Ansys, and large integration time step sizes used by the simulation tool are sufficient to capture the induced thermal and plastic strains in the model [32]. Heat flux input is not included in this modeling approach; instead, the entire activated element layer is set to the melt temperature of the material. For example, lumped layer 2 as illustrated in Fig. 5, the heat transfer between activated layers occurs via conduction with the substrate acting as a heat sink. It is assumed that complete melting of the activated layer takes place and that the process parameters for the build are appropriately set such that the occurring temperature is always at or above melt, causing no lack of fusion, and that the temperature does not far exceed melt for keyholing to occur. Aspects in SLM process such as powder behavior, and the powder-to-solid transformation is not considered in this FEM approach as the material is assumed to be in a solid state. Due to the lack of a molten pool, the convective heat transfer process in the molten liquid and, consequently, the large temperature gradients brought on by the quickly moving heat source, are ignored [18]. Furthermore, the scanning patterns which are known to have an impact on the residual stress evolutions [37] are not considered due to the associated high computational cost of simulating scanning patterns. Although the lumped layer-wise method of activation accounts for recurring heating and cooling cycles, these approximations result in less accurate thermal history and hence affects the accuracy of the estimated residual stresses. The other limitations of this modelling approach are that critical defects such as porosity, poor surface characteristics and lack of powder fusion are not considered. However, the significantly shortened computational time justifies the slight inaccuracies in the estimation of distortion and part-level residual stress using the lumped layer-wise modeling approach [38].

The FEM model of the thin-wall structure consists of layered 8 noded hexahedral elements (e.g. HEX8 as per Ansys notion) with the default full integration scheme. This element type takes into consideration elasto-plasticity, stress stiffening, large deflection, and large strain capabilities and suitable for capturing mesh-independent nonlinear effects due to residual stresses [32]. Element size of ∼0.16 mm is maintained along *y*-direction so as to have a minimum of three elements through the plate thickness, which is found to be sufficient to obtain mesh-objective results. Element layer height of 0.4 mm, which represents 10 physical layers of actual powder (e.g. 40 μm is the layer thickness of powder recoated for each layer), is used for activation. This lumping of 10 physical layers is in the recommended range (10–20 layers of powder) [32] for accuracy of results and has also been adopted by similar published work on thin-walled structures [25]. Using the lumped-layer approach, a total build height of 25 mm with approximately 62 finite element layers is achieved, which corresponds to 625 physical layers of metal powder. The base plate is meshed with a coarser mesh size of 1.5 mm. The AM simulations were performed on an Intel(R) Xeon(R) 2.30 GHz workstation and using 12 cores, with runtime ranging from 2 to 3 hours depending on the various dimensions of models considered in this work.

## 3 Results and discussions

For validating the simulation framework using the layer-by-layer activation approach, the simulated residual results are compared with experimentally measured values from published literature.The benchmarked cases for the three-material grades Ti64, IN718 and SS316L are discussed in the following sections. Since a layer-wise activation method is used in the numerical analysis, process parameters such as laser power, scanning speed, hatch spacing and scanning pattern are become irrelevant and are hence not used as input parameters in these simulation.

### 3.1 Validation of residual stresses

**Fig. 7:**
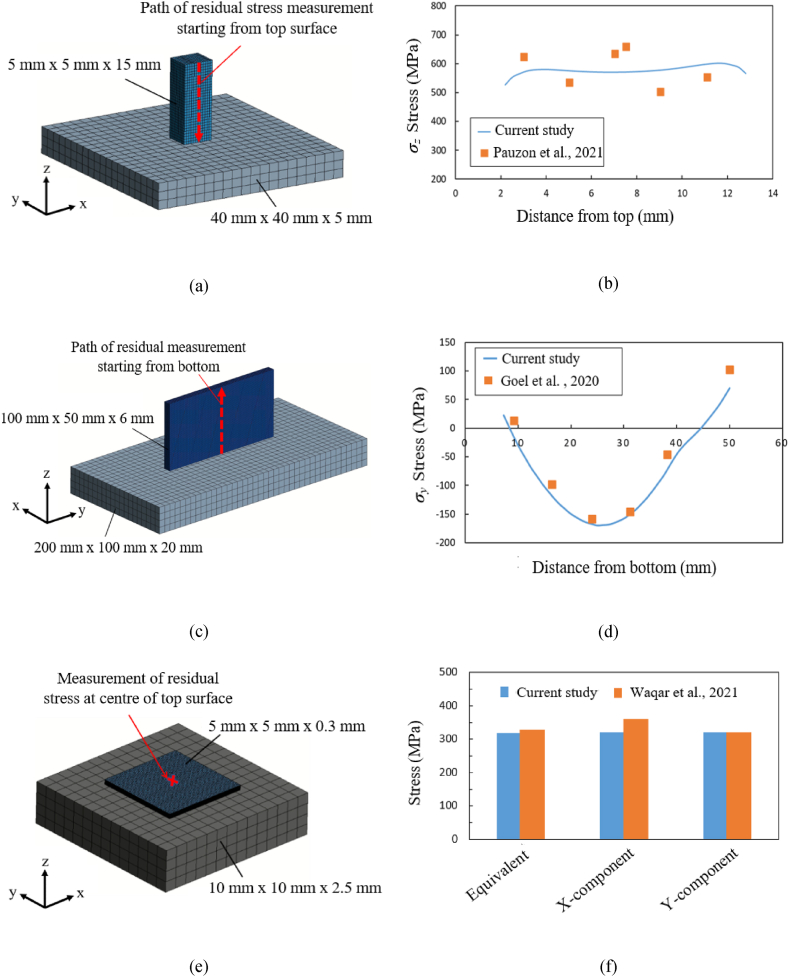
FEA models of the geometry considered for comparison with published results for three material grades; in (a) and (b) for Ti64 from Pauzon et al. [39], in (c) and (d) for IN718 from Goel et al. [40], and (e) and (f) for SS316L from Waqar et al. [41].

For the Ti64 material grade, the experimentally measured residual stress values from the work of Pauzon et al. [39] are used as a comparison with simulated residual stresses. The referenced work uses the X-ray diffraction technique for measuring the residual sample from SLM process. A cuboid sample of size 5 mm × 5 mm × 15 mm was fabricated on an EOS M290 (EOS GmbH) machine with a layer thickness of 30 μm. The residual stress measurements are conducted on the surface and along the building direction of the sample, along the centerline path indicated by the red dotted line in Fig. 7a starting from the top surface of the sample. The FEM model is shown in Fig. 7a, where element size of 0.5 mm is used for the build and a coarser mesh size of 2.5 mm is used for the baseplate (40 mm × 40 mm × 5 mm). As shown in Fig. 7b, the residual stress profile along the build direction (*z*) simulated using the layer-wise activation approach compares well with the reported experimental values.

For IN718 material, residual stress measurements using neutron diffraction method conducted by Goel et al. [40] are compared with simulated results. For the current comparison, vertical thin-wall sample of size (100 mm × 50 mm × 6 mm) from SLM process is used [40]. The sample is fabricated using EOS M290 machine with a laser power of 340 W, a scan speed of 1145 mm/s and a layer thickness of 40 μm. The residual stresses are measured along the path as indicated by the red dashed line in Fig. 7c. Fig. 7c shows FE model and geometric dimensions of the build (100 mm × 50 mm × 6 mm) and baseplate (200 mm × 100 mm × 20 mm), element layer height of 0.4 mm is used for the build and coarse mesh size of 5 mm is used for the baseplate. Residual stresses are measured on the sample after removal from the baseplate, and the simulated residual stress component *σ*_*y*_ is used for comparison with experimental values. As seen in Fig. 7d the stress profile varies from being tensile towards the wall edges to being predominantly compressive at the center of the build, and compares markedly well with the experimental results.

For the SS316L material grade, the experimental results reported by Waqar et al. [41] are used for validating the simulation results. The sample size of dimension value 5 mm × 5 mm × 0.3 mm were printed on a 3D systems (Pro-X DMP 320) SLM machine using a laser power value of 160 W, a scanning speed of 700 mm/s, and a layer thickness of 30 μm with a total of 10 build layers. The residual stresses in terms of equivalent (von Mises) stress, and the stress in *x*-component and the *y*-component are measured with X-ray diffraction technique at the center point of the top surface of the build. Fig. 7e shows the FEM model of the specimen and measurement of stress location. The build model uses a mesh size of 0.03 mm and the baseplate a coarser mesh size of 5 mm. Fig. 7f compares the computed von Mises stress and the stress components along the *x-* and *y-*axis at the center of the top surface with experimentally obtained data, showing a rather good agreement between the two.

### 3.2 Residual stresses in thin-walled structures

**Fig. 8:**
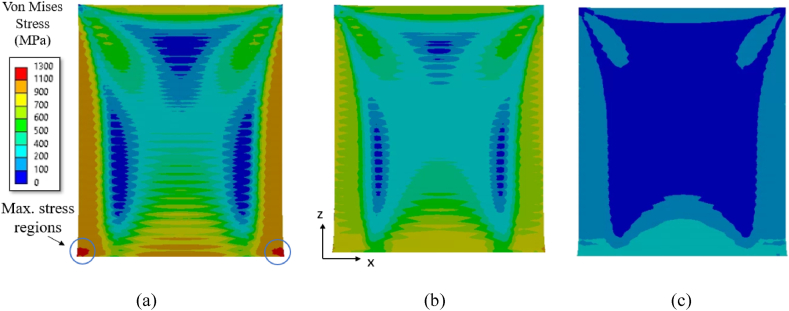
The von Mises stress plot for the thin-wall with constant thickness of 0.5 mm: (a) Ti64, (b) IN718, and (c) SS316L.

**Fig. 9:**
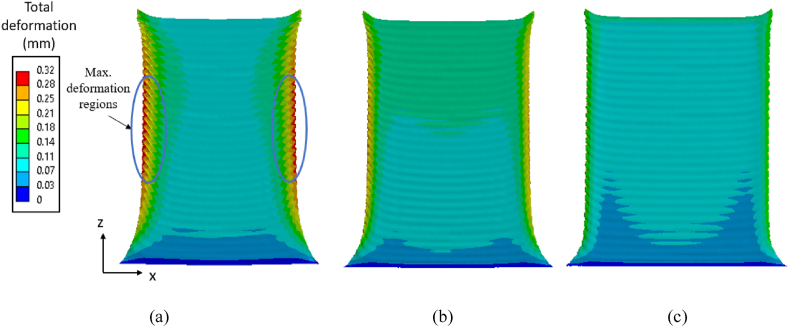
Contour plots of total deformation (magnified 10 × ) of the thin-wall after cooling to ambient temperature for: (a)Ti64, (b) IN718, and (c) SS316L.

For computing residual stresses in a thin-walled structure, the layer-by-layer build simulation as described in Section 2.3 is adopted for a thin plate of 0.5 mm thickness for the three material grades Ti64, IN718 and SS316L. The von Mises stresses after cooling the built part to room temperature for the three material grades are shown in Fig. 8a–c. Among the three material grades, Ti64 shows a higher magnitude of residual stress as compared to IN178 and SS316L, where maximum stresses are observed in regions near both ends of the interface between build and substrate. The final deformation of the thin-walled structure after cooling to ambient temperature for the three material grades is shown in Fig. 9a–c, with distortion magnified 10 times. For all cases, there is not much distortion observed at the bottom regions, i.e. near the attachment to the substrate, and at the top free surface, where it is not compressed by subsequent layers. However, the maximum distortion occurs at the middle regions of the build as this region is forced into compression because of tensile stress buildup induced by the cooling and contraction of the subsequent deposited material.

**Fig. 10:**
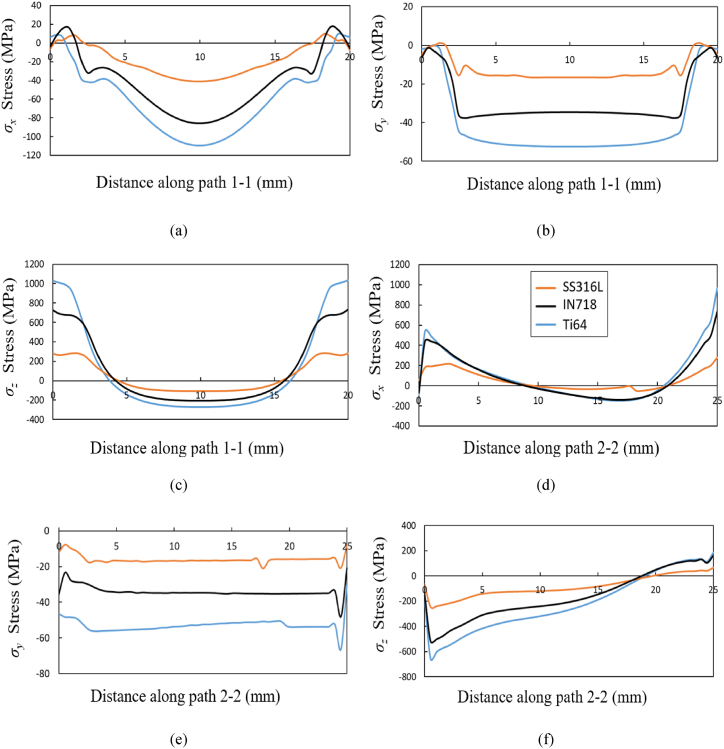
Comparison of residual stress profiles for three material grades Ti64, IN718 and SS316L and in *x,y* and *z* direction measured along path 1–1 as shown in (a), (b) and (c) respectively and along path 2–2 shown in (d), (e) and (f) respectively.

The stress components *σ*_*x*_, *σ*_*y*_ and *σ*_*z*_ in the cartesian *x,* *y* and *z* directions respectively are compared along the center line paths illustrated in Fig. 6a. The comparison of stress components for the three material grades along paths 1–1 and 2–2 are shown in Fig. 10a–c and Fig. 10d–f, indicating a highly non-uniform and mainly compressive residual stresses. Along path 1–1, the *σ*_*x*_ and *σ*_*z*_ stress tend to be tensile at the wall edges due to the wall's anchoring to the baseplate, especially in the *z* direction where rather high tensile stresses appear with values close to the yield strength of the respective material grades. These stress profiles at the center regions are similar to the observations made in published work on thin-walled structures [27]. The *σ*_*y*_ stress component for all three material grades is significantly lower than the yield strength and mostly compressive in nature. In terms of magnitude, the stress component is higher in the *z-*direction compared to the other two directions owing to the dominant path of the thermal gradient along the build height due to the layer-by-layer formation.

Similar observation of stress variation along path 2–2 can be seen, where the *σ*_*y*_ stress component is tensile near the interface of wall and substrate, while it remains comprissve at the center regions and tends to be highly tensile at the top free edge. The *σ*_*z*_ stress component is compressive at the interface of the thin-wall and the substrate, while it is tensile when approaching the free edge. These observations agree with studies found in published work on thin-walls [17]. Also, the stresses in material Ti64 are found to be high due to the lower value of thermal diffusivity (refer to Table 1) as compared to IN718 and SS316L. An additional significant factor that contributes to high residual stress is the yield strength [18], which is significantly higher at room temperature for Ti64 (1098 MPa) compared to IN718 (648 MPa) and SS316L (290 MPa).

The *σ*_*z*_ stress component along path 1–1 in Fig. 10c and *σ*_*x*_ stress component along path 2–2 in Fig. 10d are highly tensile at the free edges and may possibly lead to delamination of layers. The *σ*_*y*_ stress component seen in Fig. 10b and e for all material grades is smaller in magnitude as compared to the yield strength due to lower temperature gradient in *y* direction. Hence, the *σ*_*y*_ stress component will not be considered for further investigation henceforth in this study.

### 3.3 Effect of taper on the residual stresses

**Fig. 11:**
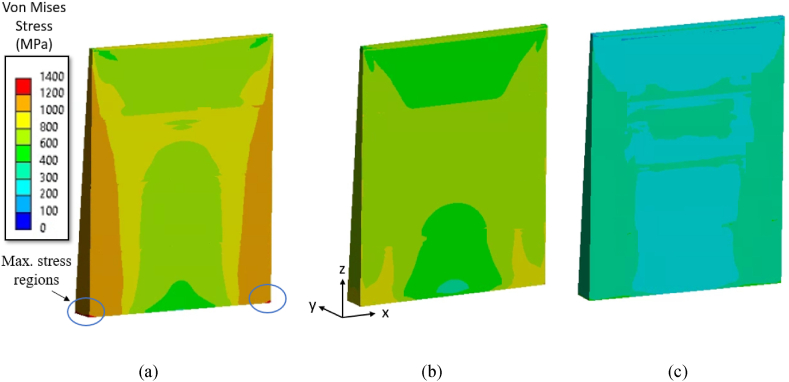
von Mises residual stress contours on a tapered thin-walled structure with bottom thickness of 2.5 mm after cooling: (a) Ti64, (b) IN718, and (3) SS316L.

**Fig. 12:**
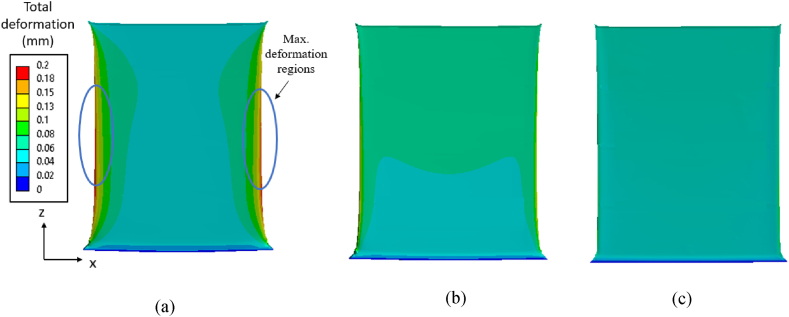
The maximum deformation (magnified 10×) on tapered wall with bottom thickness *t*_*2*_ = 2.5 mm: (a) Ti64, (b) IN718, and (c) SS316L.

The influence of tapering on the residual stress evolution is investigated by varying the bottom thickness of the thin-wall. The von Mises stress contours for a case with bottom thickness of 2.5 mm is shown in Fig. 11a–c for the three material grades. As expected, the maximum stress appears in regions near the interface of thin-wall and substrate for the three material grades. Also, the deformation pattern, as seen Fig. 12a–c, is similar to a thin-walled structure of constant thickness discussed in the previous section with maximum deformations occurring at the center regions, while the magnitude is slightly lesser owing to an increase in bottom thickness.

**Fig. 13:**
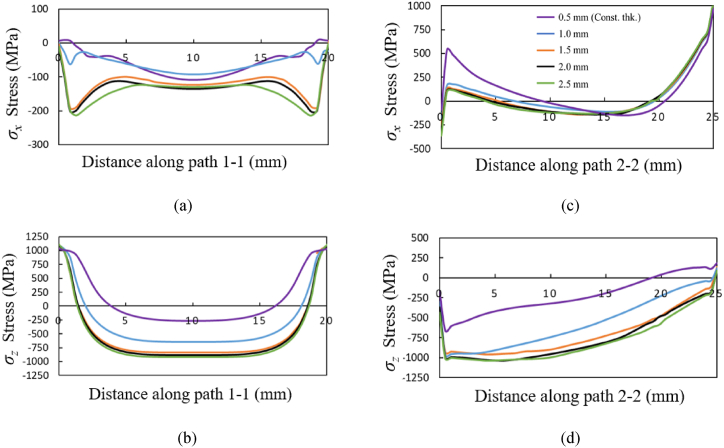
Residual stress profile in *x* and *z* direction for material grade Ti64 along path 1-1 as shown in (a) and (b) respectively and along path 2-2 shown in (c) and (d) respectively.

**Fig. 14:**
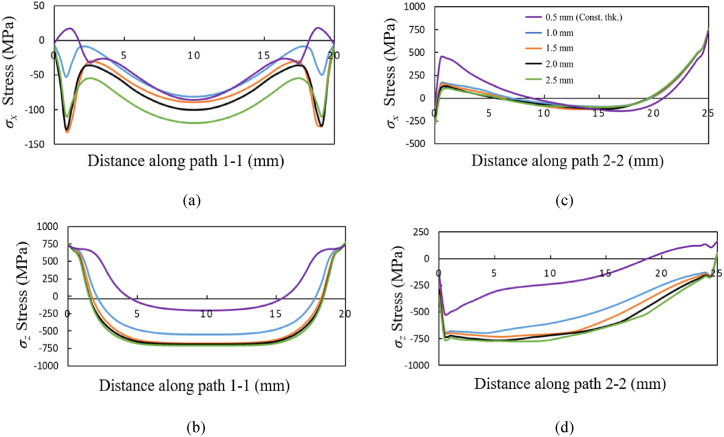
Residual stress profile in *x* and *z* direction for material grade IN718 along path 1-1 as shown in (a) and (b) respectively and along path 2-2 shown in (c) and (d) respectively.

**Fig. 15:**
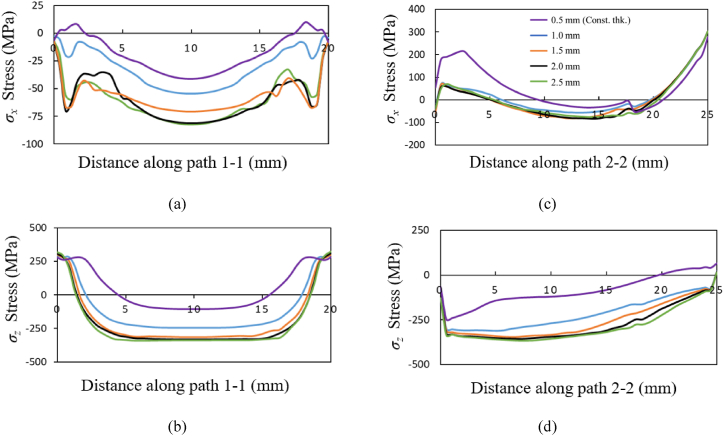
Residual stress profile in *x* and *z* direction for material grade SS316L along path 1-1 is shown in (a) and (b) respectively, and along path 2-2 shown in (c) and (d) respectively.

The stress components *σ*_*x*_ and *σ*_*z*_ along the path 1-1 and path 2-2 for the three material grades Ti64, IN718 and SS316L are shown in Fig. 13, Fig. 14, Fig. 15, respectively. The *σ*_*x*_ component of residual stress along path 1-1 and path 2-2 does not vary much with increased taper as observed from Fig. 13 (a) and (c), Fig. 14 (a) and (c), and Fig. 15 (a) and (c), where the *σ*_*x*_ stress is predominantly compressive and tending towards tensile at the free edges. However, the *σ*_*z*_ stress along path 1-1 and path 2-2 seen in Fig. 13 (b) and (d), Fig. 14 (b) and (d), Fig. 15 (b) and (d) shows an increasing trend with an increase of taper value as compared to constant wall thickness but converges for higher values of taper. For example, for bottom thickness of 1.5 mm and beyond there is no significant increases in the magnitude. The stress components *σ*_*z*_ is more dominating as compared to the *σ*_*x*_ due to the layer-by-layer build-up along the height that results in higher thermal gradients. The stress profile of *σ*_*z*_ along path 1-1 is compressive at the middle regions, where it tends towards tensile stress values at the edges and reaches the yield strength of the material grades. Along the path 1-1, *σ*_*z*_ varies from compressive, from the bottom anchored edge, to tensile at the top free surface owing to a difference in strain rates. The magnitude of residual stress generated in the tapered thin-wall are larger in magnitude as compared to the thin-wall of constant thickness, while the stress profiles are mostly similar as seen in Figs. 13, Fig. 14, Fig. 15.

### 3.4 Discussions

The residual stress estimated using the lumped layer-wise activation approach compares well with experimental measurements of SLM specimens discussed in section 3.2, and the simulated stress profiles in thin-walled specimens are similar to the observations made in the various published works mentioned in section 3.3, thus indicating that the lumped layer modeling method is suitable for quickly simulating part-level distortions and residual stress in AM processes, e.g. SLM process of SS316L using CO_2_ lasers, a most widely used industrial laser systems [42].

The stress profile in the thin-walled structure tends to be predominantly compressive at the central regions and tensile at the free edges. The tensile stress along the build direction (*z*) exhibits steep gradients at both free ends of the specimen, and the magnitudes of stress are almost equal to the yield strength for Ti64 or exceeding the yield strength in the case of IN718 and SS316L specimens, thus making it susceptible to warping and delamination of layers. However, these macroscopic stress magnitudes may be overpredicted in the current FEM model as it does not incorporate the convective heat transfers in the molten pool leading to the computation of higher temperature gradients and hence higher residual stresses [43]. The substrate has considerable influence on the distortion pattern of the build, and it is observed to be minimum near the bottom layers of the build due to proximity with the substrate which balances out the highly tensile stress occurring in the deposited layer. As the deposition progresses the influence of the substrate on subsequent layers decreases leading to a maximum contraction in the middle regions. Also, distortion at the top is minimum due to the absence of subsequent layers and no associated shrinkage.

## 4 Application to Schwarz-Primitive TPMS

**Fig. 16:**
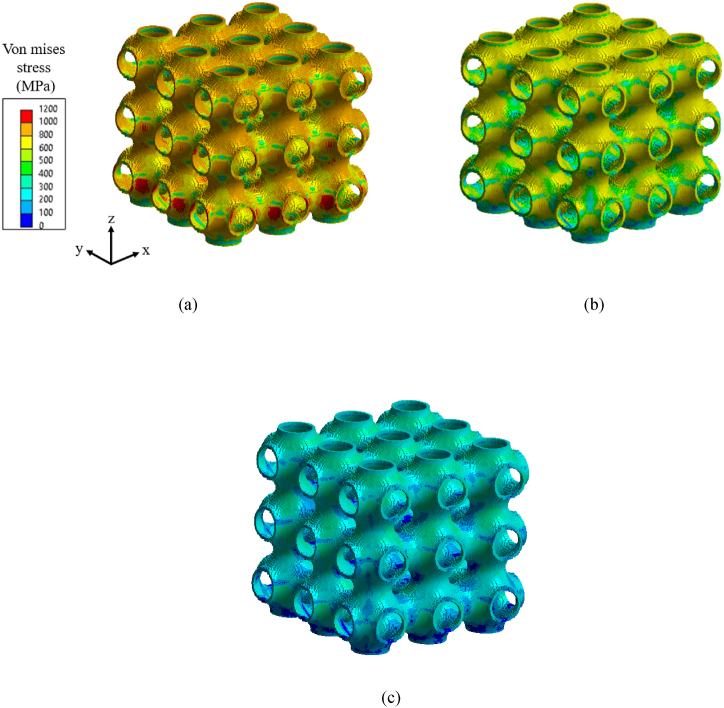
von Mises residual stress field of SP lattice configurations for the three material grades after cooling of the build to ambient temperature: (a) Ti64, (b) IN718, and (c) SS316L.

**Fig. 17:**
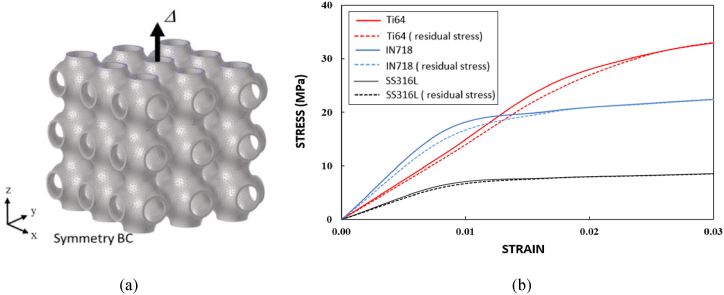
(a) FEM model of SP with uniaxial tensile loading and symmetric boundary condition (BC), (b) stress-strain curve for the three material grades in tension with and without considering the residual stresses.

The proposed simulation methodology can be applied to investigate the effect of residual stress on the mechanical behavior of complex thin-wall structures such as TPMS lattice structures built from the AM process. Based on the work by Ahmed et al. [44]*,* a 3 × 3 × 3 cell configuration of Schwarz Primitive (SP) lattice with relative density of 20%, a unit cell size of 10 mm and a wall thickness value of 1.75 mm is used. The effect of residual stress on the mechanical characteristics of the three material grades Ti64, SS316L and IN718 is investigated. The AM process parameters and FEM modeling details have been referred from published work by Ahmed et al. [44], the SP lattice is modelled with hexahedral elements (e.g. HEX8) with an element layer height of 0.4 mm according to the recommended size range [32] for this work. A total build height of 30 mm will have 75 element activation layers, which represents 750 physical layers of metal powder. The base plate (50 mm × 50 mm × 10 mm) uses a coarser mesh size of 5 mm. Fig. 16a–c shows the residual stress distribution of the build after cooling to room temperature (22 °C) for the three material grades. As observed in earlier sections, the magnitude of residual stress is greater in the Ti64 structure than in the SS316L and IN718 structures. The final residual stress state from the AM simulation is then utilized to analyze the part's effective mechanical properties, such as elastic modulus and yield strength under tensile loading. Fig. 17a illustrates the tensile loading situation for an SP lattice structure along with symmetric boundary conditions in the *z*-direction applied on the base plane. All six of the stress tensor components from AM thermal simulation are mapped onto the FE model's nodes as initial conditions in the mechanical simulation. The top surface of the lattice is subjected to a displacement load with a magnitude of 0.3 mm in tension along the build direction, resulting in a total normal average strain of 10%. Fig. 17b displays the uniaxial tensile stress-strain curves for each of the material grades, considering both the effects of residual stress from the AM process and without including residual stress effects. The stress-strain curves show that residual stress has a noticeable effect on the elasticity of the structures, with a reduction in elastic mofulus ranging from 6% to 10% for the various material grades. The presence of residual stress, on the other hand, has no effect on the mechanical response of the lattice structures in the plastic region for any of the material grades. Thus, by adopting the current simulation framework we can investigate the effect of inherited residual stresses on the overall mechanical properties of sheet or shell-based lattice metamaterials. In these simulations, it is shown that the residual stresses are mainly affecting the elastic modulus of the lattice. Future work should investigate the effect of the residual stresses on deformation behavior and fracture of these sheet or shell-based lattices.

## 5 Conclusions

Numerical investigation using layer-wise activation approach is undertaken for investigating the residual stress profile in a thin-walled structures using three different material grades Ti64, IN718 and SS316L. The residual stresses at the center of the thin-walled structures are found to be predominantly compressive while at the free edges are highly tensile and found to be almost equal or beyond the yield strength of the materials. The deformation pattern takes an hourglass shape, owing to the influence of substrate at the bottom layers and absence of subsequent deposition at the top surface. Deformation at the central regions of the wall was found to be maximum due to cooling and contraction of the subsequent layers deposited. Also, the effect of varying the tapering on residual stress is studied and observed that for an increasing taper there is an increase in residual stress in the build direction and converges beyond a certain value of taper, i.e. for bottom thickness value of 1.5 mm. The magnitude of residual stress along the build height direction is comparatively higher indicating thermal gradient is more dominant along the height due to the layer-wise formation. The residual stress in Ti64 samples is higher compared to that in IN718 and SS316L due to the higher yield stress at room temperature and lower diffusivity value of Ti64 when compared with IN718 and SS316L. The proposed simulation framework can be also applied to complex thin-walled structures such TPMS lattice structures to investigate the effect of residual stress on their mechanical properties. It is observed that the residual stresses have a significant impact on the effective elastic modulus of these TPMS structures, where a reduction in stiffness was observed in the range of 6%∼10% for the three material grades. However, these inherited residual stresses have an insignificant impact on the plastic behavior of the structures.

The computed residual stress data can be used in future work to examine the warpage, layers delamination and bending in thin-walled structures. The findings may provide a better understanding of stress distribution during the powder bed process and help in process parameter optimization for fabricating dimensionally conforming thin-wall structures. The study can be further expanded by considering the use of support structures for better heat dissipation and design applications of thin-walled structures where mitigation of residual stress is desired.

## Declarations

### Author contributions statement

1 - Conceived and designed the experiments.

2 - Performed the experiments.

3 - Analyzed and interpreted the data.

4 - Contributed reagents, materials, analysis tools or data.

5 - Wrote the paper.

### Data availability statement

Data included in article/supp. material/referenced in article.

## Declaration of competing interest

Authors hereby declare that there is no conflict of interest.
